# Artificial Selection Trend of Wheat Varieties Released in Huang-Huai-Hai Region in China Evaluated Using DUS Testing Characteristics

**DOI:** 10.3389/fpls.2022.898102

**Published:** 2022-06-10

**Authors:** Liyuan Wang, Yongsheng Zheng, Lili Duan, Mumu Wang, Hui Wang, Hua Li, Ruyu Li, Han Zhang

**Affiliations:** Crop Research Institute, Shandong Academy of Agricultural Sciences, Jinan, China

**Keywords:** wheat varieties, DUS testing characteristics, Huang-Huai-Hai region, artificial selection trend, variation diversity

## Abstract

Wheat has been widely cultivated all over the world. In China, the number of approved wheat varieties has steadily grown since 2010, with the most notable trend in the Huang-Huai-Hai region. Distinctiveness, uniformity, and stability (DUS) are the prerequisites for a new wheat variety to obtain a release permit. Yet, few reports are available on DUS testing characteristics of released wheat varieties. Here, 32 DUS testing characteristics of 195 wheat varieties released in the Huang-Huai-Hai region were investigated to study their artificial selection trend. The results showed that the means, ranges, and coefficients of variation for eight measured characteristics varied greatly, among which the number of sterile spikelets had the largest variation coefficient of all three wheat-growing areas in the Huang-Huai-Hai region. The difference in plant height between the three wheat-growing areas was the most significant. The mean plant height in the northern winter wheat area was the largest, while that in south Huanghuai was the smallest. The released varieties of the three wheat-growing areas in the region had similar artificial selection trends in some characteristics. For instance, flag leaf length and flag leaf width, grain number per ear, and grain volume weight showed an overall upward trend, while the plant height gradually decreased. The clustering results based on DUS testing characteristics showed that artificial selection of characteristics was consistent with ecological adaptation and breeding process as well as pedigree sources. Our findings indicated that with the current breeding objectives, the selection of some non-economic characteristics of wheat varieties, such as awn color, stem color, and glume color, seemed to be able to enrich the genetic diversity of varieties in the Huang-Huai-Hai region. These results could provide guidance for subsequent wheat breeding and production in this region, screening similar varieties, and determining the distinctness of applied varieties in DUS testing.

## Introduction

Wheat is widely cultivated in the world, and it is also one of the three major grain crops in China. According to the National Bureau of Statistics, the sown area and output of wheat accounted for 20.44 and 20.13% of the total crop sown area and output of grain crops in China, respectively (2019). In China, the Huang-Huai-Hai region is the main wheat-growing area, accounting for 57.54 and 63.10% of the total wheat sown area and output, respectively. The Huang-Huai-Hai region is a large eco-geographical zone of about 469,500 square kilometers, roughly covering areas of Beijing, Tianjin, Hebei, Shandong, and Henan provinces. It can be divided further into three wheat-growing areas: the northern winter wheat area (Z1), the north Huanghuai winter wheat area (Z2) and the south Huanghuai winter wheat area (Z3).

The goal of wheat breeding is related to the development, orientation, and practical demand of the market economy (Han et al., 2017). As the breeding objectives change at the different stages, the traits of wheat varieties bred and marketed at the stages are different. Analyzing and summarizing the trend patterns and directions of wheat varieties can provide guidance for subsequent wheat breeding and production. At present, there have been a number of reports on the evolution of agronomic traits of wheat varieties in different wheat areas. For instance, in a report on winter wheat in America great plains, the authors investigated the yield attributes of 14 varieties introduced between 1873 and 1994 (Donmez et al., 2001). Their results suggested that the reduction of height, early maturity, and resistance to lodging contributed to the upward trend of yield. An analysis on winter wheat cultivars planted in France from 1946 to 1992 showed that the decrease of height played the most important role in winter wheat yield, followed by the ability to use the total above-ground biomass to produce more grains (Brancourt-Hulmel et al., 2003). An experiment using 117 winter wheat cultivars released between 1920 and 2000 to appraise the changes in various agronomic traits in Chile found that with the change of certification time, the harvest index increased and the plant height decreased gradually (Matus et al., 2012). The study on the evolution of wheat cultivars released in China also indicated increasing grain weight and yield with decreasing plant height and maximum tillers (Song et al., 2013).

The number of wheat varieties used in the aforementioned research ranged from dozens to hundreds. And the research objects mostly involved yield and yield-related elements. In many countries, distinctness, uniformity, and stability are the prerequisites for a new plant variety to obtain protection and registration (Deng et al., 2019). To meet the DUS criterion, a new wheat variety must be shown clearly different from all other varieties of common knowledge, reasonably uniformity and stability based on several of phenotypic characteristics (Wang et al., 2002). The phenotypic characteristics are listed in wheat test guidelines and range from morphological, phonological to agronomic traits. In the above-mentioned studies, the authors used almost exclusively yield and yield-related traits. Studies based on the DUS testing characteristics are reported rarely. Summarizing and analyzing the characteristics related to DUS testing can not only evaluate the ability of these characteristics to distinguish varieties, but also provide supplementary information for new variety breeding (Wang et al., 2002; Reddy et al., 2009; Singh et al., 2012; Jones et al., 2013; Pilarczyk et al., 2015). In this study, 195 wheat varieties of the Huang-Huai-Hai origin were used as materials and 32 characteristics listed in China national test guidelines for wheat were observed and analyzed. The artificial selection trend of the varieties was analyzed in chronological order. We hope the results from this study can play a guiding role in the selection and cultivation of wheat varieties from the aspect of new variety approval.

## Materials and Methods

### Plant Materials

The 195 wheat varieties used in this study were released in the Huang-Huai-Hai region in the past 20 years (Supplementary Table 1). The information on release time, suitable planting areas, and pedigrees were derived from the Data Platform of China Seed Industry (http://202.127.42.145/home/manageorg). There were 34, 79, and 82 varieties from the northern winter wheat area, the north Huanghuai winter wheat area and the south Huanghuai winter wheat area, respectively.

### Method of Examination for DUS Testing Characteristics

The materials were planted in the experimental base of the Crop Research Institute, Shandong Academy of Agricultural Sciences (Jinan, China) in October 2019 and 2020. The experimental design was a 6-row area with a row length of 6 m and a row spacing of 0.25 m, with two repetitions.

The characteristics investigated were those listed in the national DUS test guidelines for common wheat, including a total of 32 qualitative characteristics (QL), pseudo-qualitative characteristics (PQ), and quantitative characteristics (QN). The detailed information on the 32 characteristics were listed in Table 1. The observations were made in accordance with the wheat test guidelines. The observation methods of characteristics included visual assessment by a single observation of a group of plants or parts of the plant (VG), measurement of a number of individual plants or parts of plants (MS), and a single measurement of a group of plants or parts of plants (MG). The corresponding codes of visually assessed characteristics and expression status were recorded according to the guidelines. For each measured characteristic, such as flag leaf length, flag leaf width, plant height, ear length, spikelet number per ear, sterile spikelet number per ear, and grain number per ear, at least 20 typical strains were selected, measured, and recorded one by one for each variety. The grain volume weight was measured by HGT-1000A in accordance with the guidelines (GB/T 5498-2013).

**Table 1:** The information on wheat DUS testing characteristics used in this study.

**Characteristics**	**Character code**	**Type of expression**	**Method of observation**	**States and code of expression**
Coleoptile: anthocyanin coloration (CAC)	char1	QN	VG	absent or very weak (1); weak (2); strong (3)
Plant: growth habit (PGH)	char2	QN	VG	Erect (1); semi erect (3); prostrate (5)
Leaf: intensity of green color (LIGC)	char3	QN	VG	light (3); medium (5); dark (7)
Anthocyanin coloration of auricles (ACA)	char5	QN	VG	absent or very weak (1); weak (2); strong (3)
Time of ear emergence (TEE)	char6	QN	VG	very early (1); early (3); medium (5); late (7); very late (9)
Flag leaf sheath wax (FLSW)	char7	QN	VG	absent or very weak (1); weak (3); medium (5); strong (7); very strong (9)
Ear wax (EW)	char8	QN	VG	absent or very weak (1); weak (3); medium (5); strong (7); very strong (9)
Stem wax (SW)	char9	QN	VG	absent or very weak (1); weak (3); medium (5); strong (7); very strong (9)
Flag leaf length (FLL)	char10	QN	MS	short (3); medium (5); long (7)
Flag leaf width (FLW)	char11	QN	MS	narrow (3); medium (5); broad (7)
Ear exsertion (EE)	char12	PQ	VG	very short (1); short (3); medium (5); long (7); very long (9)
Plant height (PH)	char13	QN	MS	very low (1); low (3); medium (5); high (7); very high (9)
Ear length (EL)	char14	QN	MS	very short (1); short (3); medium (5); long (7); very long (9)
Spikelet number per ear (SNPE)	char15	QN	MS	few (3); medium (5); many (7)
Sterile spikelet number per ear (SSNPE)	char16	QN	MS	absent or very few (1); few (3); medium (5); many (7)
Grain number per ear (GNPE)	char17	QN	MS	very few (1); few (3); medium (5); many (7); very many (9)
Ear density (ED)	char18	QN	VG	lax (3); medium (5); dense (7)
Ear shape (ES)	char19	PQ	VG	fusiform (1); conical (2); ellipse (3); rectangle (4); stick shape (5); branched (6)
Awn type (AT)	char20	PQ	VG	absent (1); straight (2); curved (3)
Awn length (AL)	char21	QN	VG	very short (1); short (3); medium (5); long (7); very long (9)
Awn color (AC)	char22	QL	VG	light yellow (1); red (2); black (3)
Stem color (SC)	char23	QL	VG	yellow (1); purple (2)
Straw: pith in cross section (SPCS)	char24	QN	VG	absent or thin (1); medium (2); thick or filled (3)
Glume color (GLC)	char25	PQ	VG	yellow (1); red (2); black (3)
Glume hairiness (GLH)	char26	QN	VG	less (3); medium (5); multiple (7)
Glume shape (GLS)	char27	PQ	VG	ovate (1); near circular (2); ellipse (3); oblong (4)
Glume: shoulder shape (GLSS)	char28	QN	VG	inclined shoulder (1); square shoulder (2); shoulder (3)
Glume: length of beak (GLLB)	char29	QN	VG	very short (1); short (3); medium (5); long (7); very long (9)
Grain shape (GS)	char30	PQ	VG	oblong (1); oval (2); ellipse (3); near circular (4)
Grain texture (GT)	char33	QN	VG	silty (1); semi-horny (3); horny (5)
Seasonal type (ST)	char34	PQ	VG	spring type (1); partial spring type (2); semi-winter type (3); partial winter type (4); winter type (5)
Grain volume weight (GVW)	char41	QN	MG	very low (1); low (3); medium (5); high (7); very high (9)

*QL, Qualitative characteristic; QN, Quantitative characteristic; PQ, Pseudo-qualitative characteristic; MS, measurement of a number of individual plants or parts of plants; MG, single measurement of a group of plants or parts of plants; VG, visual assessment by a single observation of a group of plants or parts of plants*.

### Statistical Analysis of Phenotypic Data

For the visually assessed characteristics, the observed codes across 2 years were sequentially corrected by the standard varieties. For each measured characteristic, means of 40 typical strains aggregated over 2 years were used for the statistical analysis. The data of each characteristic was analyzed by SPSS 21.0 (IBM Corp., Armonk, NY) for descriptive statistics and correlation. According to the release time, the wheat varieties were divided into four groups to analyze the artificial selection trends of DUS testing characteristics. The phenotypic distances of wheat varieties were carried out through R packages ‘dist': Distance Matrix Computation. First, the data for each characteristic were standardized by dividing by the sample SD to remove the effect of dimensionality. Then, the various distances were calculated according to Euclidean and clustered using Ward's clustering algorithm. The graphics were drawn by the ‘ggplot2' package in R software or GraphPad Prism to visualize the results. The dendrogram was drawn and beautified using the online tool EvolView (https://www.evolgenius.info/evolview/).

## Results

### Observation and Analysis of DUS Testing Characteristics

**Figure 1:**
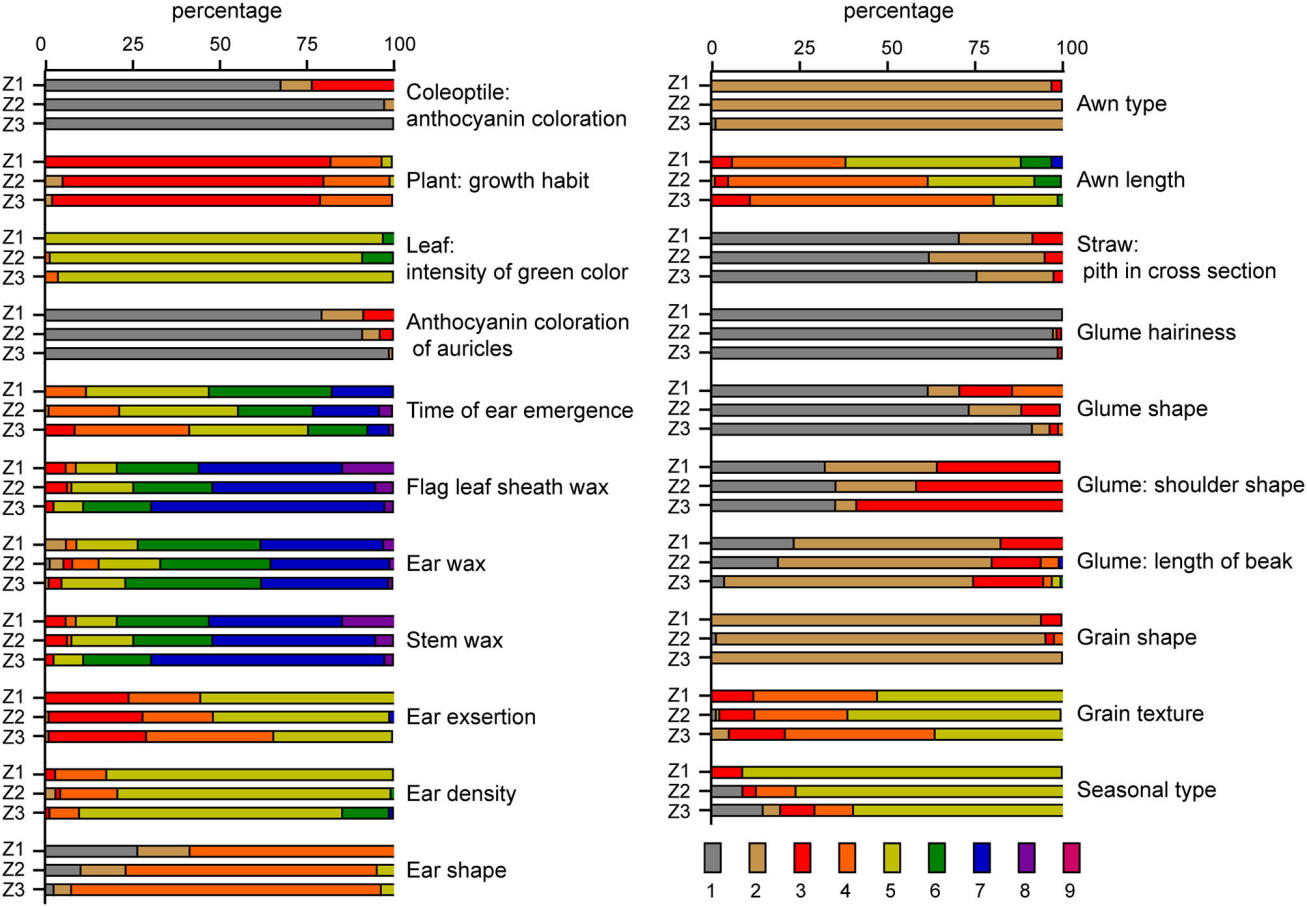
Distribution of variation types of 21 visually assessed characteristics in 195 wheat varieties. Z1, Z2 and Z3 represent the northern winter wheat area, the north Huanghuai winter wheat area and the south Huanghuai winter wheat area respectively. Numbers 1–9 are the corresponding expression status codes assigned to the visually assessed characteristics according to the guidelines. The specific meaning of each characteristic and observation code is listed in Table 1.

In total, 32 characteristics of 195 varieties from the three wheat-growing areas in the Huang-Huai-Hai region were observed and analyzed. There was only one state of expression observed for characteristics awn color, stem color, and glume color in all the tested 195 varieties. Therefore, they would not be analyzed further. The results of the remaining 21 visually assessed characteristics were shown in Figure 1. For the straw pith in cross-section and glume shoulder shape, varieties from the three wheat-growing areas all had the same spectrum of expression states but different distribution frequencies for these states. For the other 19 visually assessed characteristics, varieties from the three wheat-growing areas showed clear differences in both the spectrum of expression states and distribution frequencies for these states. For the coleoptile anthocyanin coloration, leaf intensity of green color, anthocyanin coloration of auricles, awn type, and glume hairiness, varieties from the three wheat-growing areas shared the same major state of expression and had <3 states of expression. There were four or more states of expression for the time of ear emergence, flag leaf sheath wax, ear wax, stem wax, ear density, awn length, glume shape, and seasonal type in all three groups of varieties. Overall, the 195 varieties from the three wheat-growing areas displayed the rich genetic diversity.

The descriptive statistical results of the eight measured characteristics for varieties from three growing areas were shown in Table 2. The mean, range, and coefficient of variation (CV) of the characteristic for each growing area differed from each other. Sterile spikelet number per ear had the highest CV among the eight characteristics with the CV of the varieties in Z2 being the largest. Grain volume weight had the smallest CV among the eight characteristics, with the varieties from Z3 showing the lowest CV among the three wheat-growing areas. The differences between the three growing areas on flag leaf width, plant height, sterile spikelet number per ear, and grain number per spike were significant (*p* < 0.05).

**Table 2:** Statistical analysis results of eight measured characteristics of 195 wheat varieties.

**Characteristics**	**Suitable planting areas**	**Mean^a^**	**STDEV**	**Min.**	**Max.**	**Range**	**C.V./%**
Flag leaf length (FLL)	Z1	19.76a	2.6	14.5	28.13	13.64	13.14
	Z2	19.66a	2.37	14.64	28.02	13.38	12.04
	Z3	20.38a	2.25	13.78	25.89	12.11	11.06
Flag leaf width (FLW)	Z1	1.75b	0.2	1.42	2.08	0.66	11.19
	Z2	1.82b	0.2	1.2	2.51	1.31	11.16
	Z3	1.91a	0.23	1.54	3.01	1.47	11.98
Plant height (PH)	Z1	78.66a	7.53	65.67	98.33	32.67	9.57
	Z2	72.43b	7.43	53	97.67	44.67	10.26
	Z3	65.49c	6.09	50.67	79.35	28.68	9.3
Ear length (EL)	Z1	9.07a	0.77	7.38	11.83	4.46	8.45
	Z2	9.06a	0.92	7.04	12.27	6.35	10.15
	Z3	8.85a	0.65	7.51	11.9	4.39	7.29
Spikelet number per ear (SNPE)	Z1	18.02a	1.28	16	20.85	4.85	7.08
	Z2	18.18a	1.41	16.42	20.65	10.18	7.64
	Z3	18.37a	1.06	16.2	21.25	5.05	5.75
Sterile spikelet number per ear (SSNPE)	Z1	0.75b	0.47	0	1.75	1.75	63.3
	Z2	0.66b	0.51	0	2.1	2.1	76.93
	Z3	0.86a	0.45	0.05	1.95	1.9	52.05
Grain number per ear (GNPE)	Z1	50.39ab	4.71	43.8	64.55	20.75	9.35
	Z2	52.95a	6.14	38.8	73.3	34.5	11.6
	Z3	53.17a	4.49	40.05	64.8	24.75	8.44
Grain volume weight (GVW)	Z1	783.79a	39.56	709	838	129	5.05
	Z2	798.35a	31.57	706	842	136	3.95
	Z3	795.69a	22.35	705	833	128	2.81

a *The various lowercase letters following the data represent significant differences at a 0.05 level based on one-way ANOVA*.

### The Artificial Selection Trend of Tested Materials

Varieties in this study were divided into four groups according to their approved time. Groups I, II, III, and IV each consisted of 22 varieties registered before 2000, 65 varieties registered between 2001 and 2010, 60 varieties registered between 2011 and 2015, and 48 varieties registered since 2016. The information on registration time and suitable planting areas for the varieties is listed in Supplementary Table 1.

**Figure 2:**
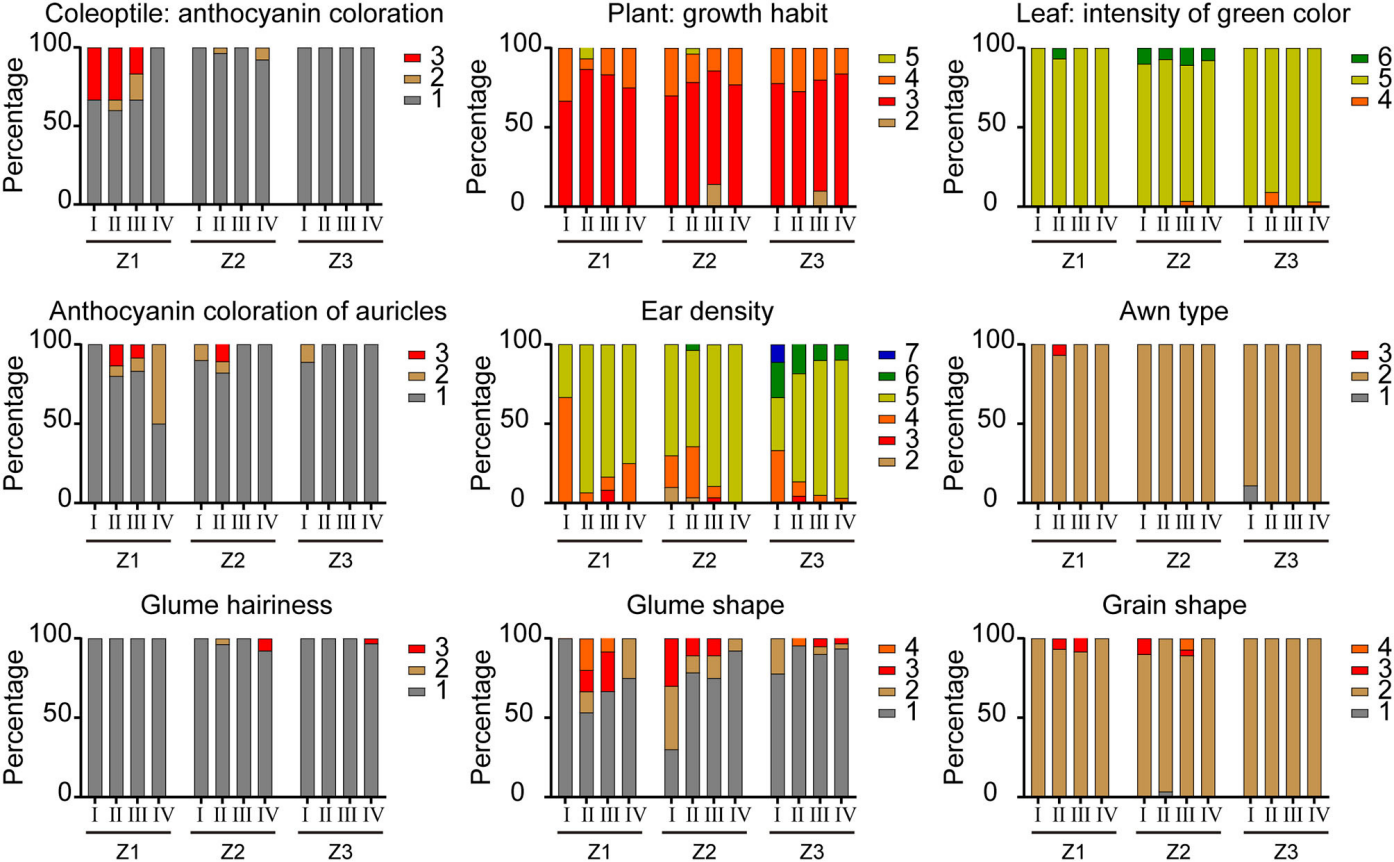
Changes in nine visually assessed characteristics of wheat varieties with the first artificial selection trend. Roman numerals I, II, III and IV refer to different groups according to the approved time. The rest of the legends have the same meaning as in Figure 1.

**Figure 3:**
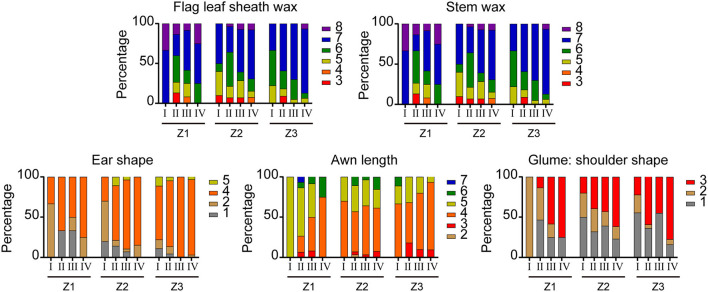
Changes in five visually assessed characteristics of wheat varieties with the second artificial selection trend. The legends have the same meaning as in Figures 1, 2.

**Figure 4:**
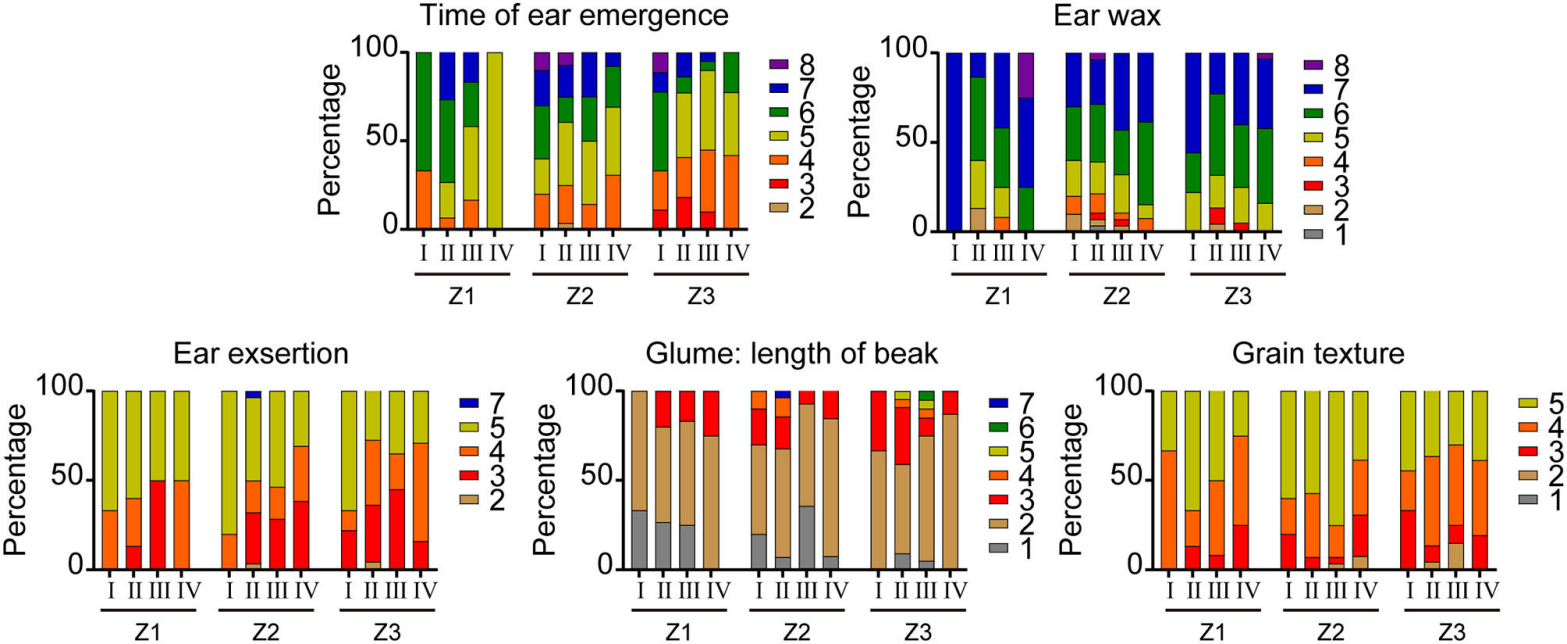
Changes in five visually assessed characteristics of wheat varieties with the third artificial selection trend. The legends have the same meaning as above.

The artificial selection trends of DUS testing characteristics of varieties from the three growing areas released at different stages were analyzed. Among the 195 varieties, the straw pith in cross-section and seasonal type had great differences between the three wheat-growing areas. With the change of years, the “absent or thin” type of straw pith became the dominant one and the spring type became the only type for varieties in the northern winter wheat area (Z1), while the expression states remained diversified for varieties in the north Huanghuai winter wheat area (Z2) and the south Huanghuai winter wheat area (Z3) on the two characteristics. The other 19 visually assessed characteristics in varieties of the three wheat areas showed similar artificial selection trends, which could be classified into three types. For type one (Figure 2), one state of expression dominated in all stages of registration. This type included nine characteristics: coleoptile anthocyanin color, plant growth habit, leaf intensity of green color, anthocyanin coloration of auricles, ear density, awn type, glume hairiness, glume shape, and the grain shape. For type two (Figure 3), the proportion of a certain state of expression increased gradually over the years and changed in a specific direction. Such characteristics included flag leaf sheath wax, stem wax, ear shape, awn length, and glume shoulder shape. The artificial selection trends of the first and second types were direct or indirect results of purposeful selection by breeders in the breeding process. For type three (Figure 4), a number of expression states (three or more) existed for the characteristics and the proportions of the expression states did not change significantly over the years. The characteristics of type three included time of ear emergence, ear wax, ear exsertion, glume length of beak, and grain texture. The stable distribution pattern of states of expression over years of type three characteristics suggested that they were not the main factors in the breeding selection process.

**Figure 5:**
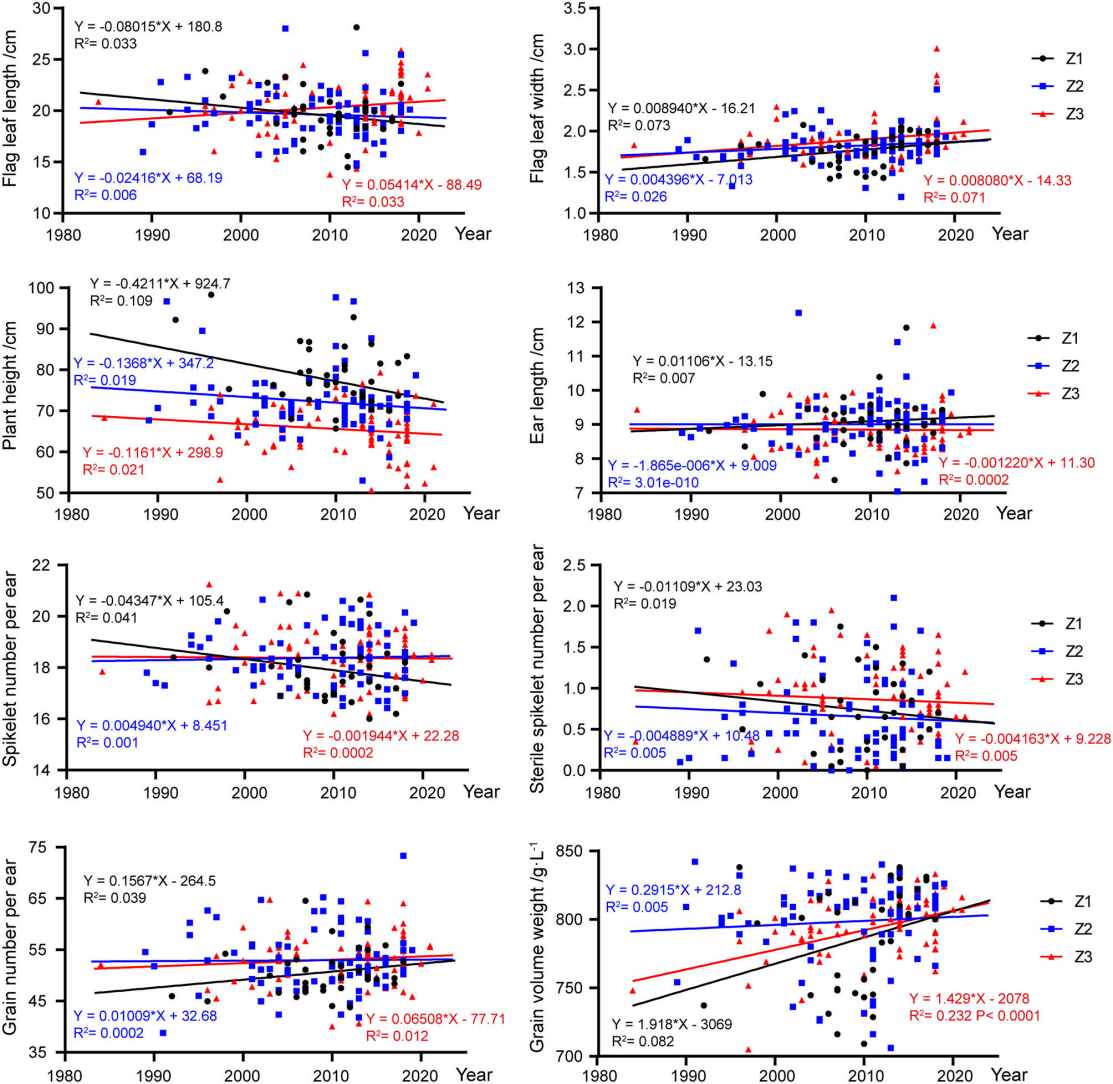
Changes in eight measured characteristics of wheat varieties in different years and wheat areas. Circles, squares, and triangles represent wheat varieties from Z1, Z2, and Z3 zones, respectively. The fitted equations for the Z1, Z2, and Z3 zones are distinguished in black, blue and red, respectively. *R*^2^ represents the regression coefficient of each regression equation.

The statistical results and trend diagrams for the eight measured characteristics of registered varieties across different periods and wheat regions were shown in Figure 5. For flag leaf length and sterile spikelet number per ear, varieties in both Z1 and Z2 showed a downward trend, although the trend for varieties in Z1 was more obvious. Flag leaf width and grain number per spike showed a growing trend, however, the increase was more pronounced for varieties in Z1 than varieties in Z2 and Z3. Ear length remained more or less stable in the three wheat-growing areas. Spikelet number per ear showed a similar downward tendency in Z1 and Z3, but the decreasing trend in Z1 was more significant. In all three areas, plant height had been declining while grain volume weight had been increasing. However, the strength of the trends over the years for varieties from the three areas were significantly different. The plant height for varieties in Z1 showed the fastest rate of decrease, while the grain volume weight increased more rapidly for varieties in Z3. For the varieties registered after 2016, the mean plant height for varieties in Z3 were significantly lower than both varieties in Z1 and Z2, while the mean grain volume weight for varieties in Z3 was the highest among varieties from the three areas.

### Correlations Between DUS Testing Characteristics

Correlation analysis among DUS testing characteristics of the 195 varieties was conducted and the correlation results were listed in Supplementary Table 2. The correlation coefficients among the three wax-related characteristics of flag leaf sheath wax, ear wax, and stem wax reached significant level. Flag leaf length had a significant positive correlation with flag leaf width. For the seven ear-related characteristics, ear density was positively correlated with ear shape and the number of grains per ear. Ear length was negatively correlated with ear shape but positively correlated with spikelet number per ear. Grain number per ear was positively correlated with ear length, spikelet number per ear, ear density, and ear shape, and was negatively correlated with sterile spikelet number per ear. For the remaining characteristics, the correlation coefficients among awn-related characteristics (awn type and awn length), glume-related characteristics (glume shape and glume length of beak) and grain-related characteristics (grain shape, grain texture, and grain volume weight) did not reach significant levels. In the combination of characteristics with significant relationships, the correlation between flag leaf sheath wax and stem wax was the strongest with a correlation coefficient of 0.99. Most of the remaining correlation coefficients ranged from 0 to 0.4, indicating that there was only a weak relationship between the relevant characteristics.

### Cluster Analysis Based on DUS Testing Characteristics and Pedigrees

**Figure 6:**
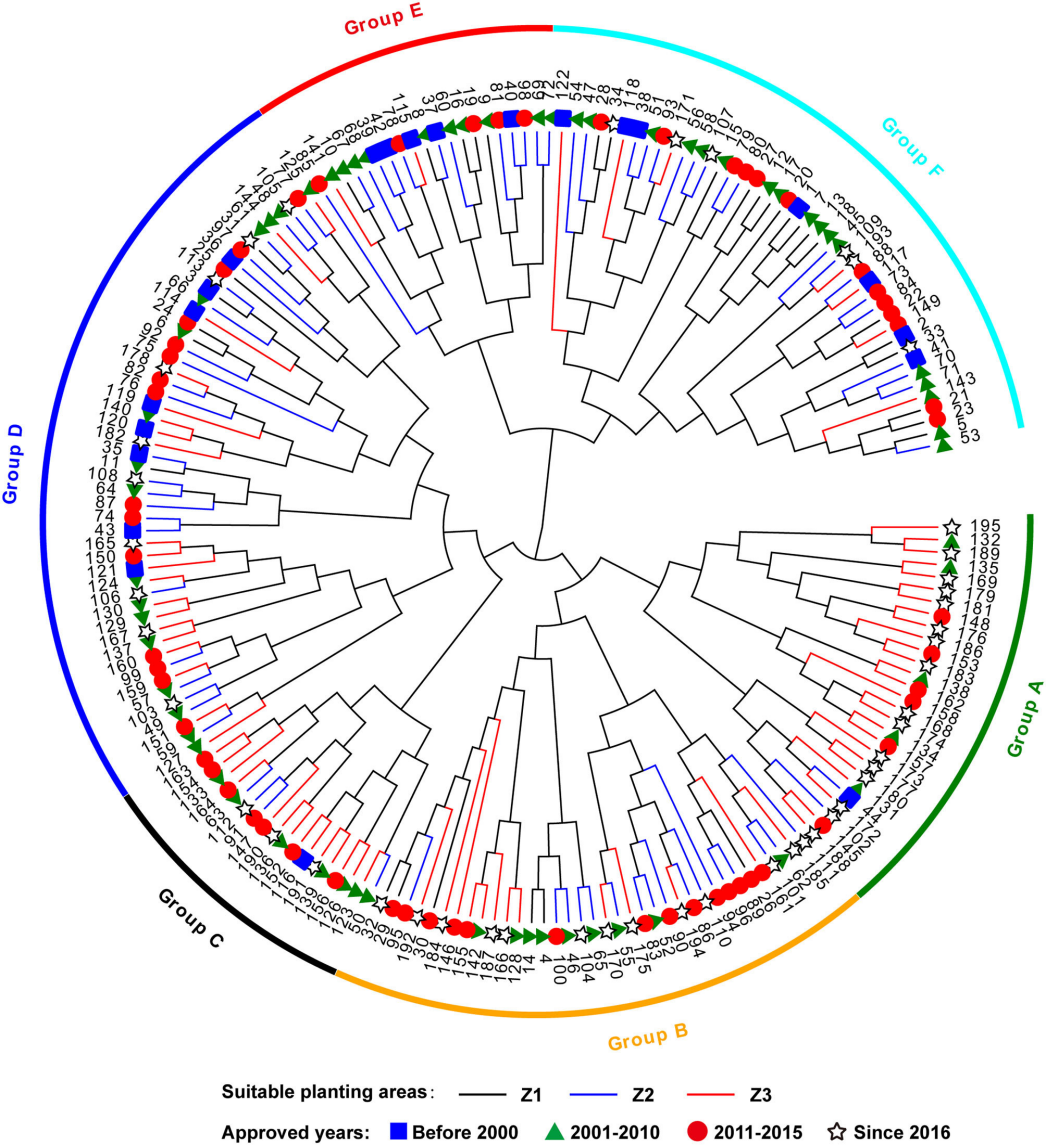
Cluster dendrogram of 195 wheat varieties based on DUS testing characteristics. The variety information corresponding to the number is listed in Supplementary Table 1. Squares, triangles, circles and stars represent wheat varieties from group I, II, III and IV according to the approved time. The black, blue and red lines represent wheat varieties from the Z1, Z2, and Z3 zones, respectively.

The 195 wheat varieties were divided into six groups according to the clustering dendrogram constructed from the DUS testing characteristics (Figure 6). Group A included 28 wheat varieties that were mainly suitable for planting in the south Huanghuai winter wheat area (Z3) and approved since 2016. Group B contained 37 wheat varieties mainly from the north Huanghuai winter wheat area (Z2) and the south Huanghuai winter wheat area (Z3). And most of the wheat varieties in Group B were released between 2011 and 2015. Group C was the smallest one, including 17 wheat varieties mainly from Z3, and released between 2000 and 2015. Group D was the largest one with 50 wheat varieties mainly from Z2 and Z3. Group E had 20 wheat varieties from the north Huanghuai winter wheat area and the approval was before 2010. Group F contained the remaining 43 wheat varieties from the northern winter wheat area (Z1) and the north Huanghuai winter wheat area (Z2). The approval time of wheat varieties in Groups D and F was mainly before 2015. From the clustering results in Figure 6, the wheat varieties in Z1 were the farthest from those in Z3. And most of the wheat varieties on the same clade were also approved during the same period. The results of this study showed that artificial selection of characteristics was consistent with ecological adaptation and the breeding process to a certain extent.

According to the information of pedigrees obtained from the Data Platform of China Seed Industry Supplementary Table 1), we found that the parents of most wheat varieties in Groups A and C were Zhoumai and Yumai series materials from the Henan Province, among which Zhoumai16 and Zhoumai22 were the most widely used parent materials. The wheat varieties in Group B were mostly derived from parent lines from Shandong and Hebei provinces. Group D contains the largest number of materials and the most extensive sources. Their parental materials came from Hebei, Shandong, and Henan provinces. Nonetheless, the materials with similar sources are more closely branched in the clustering results. Therefore, the clustering results of DUS testing characteristics were also consistent with pedigree analysis.

## Discussion

At present, most research reports on the evolution of agronomic traits of wheat varieties focused mainly on the yield and yield-related components or quality traits. In contrast to those, the characteristics specified in the DUS test guidelines of wheat were used in this study for observation and data analysis. The wheat materials used in this study were those bred and released in the Huang-Huai-Hai region in China in the last 20 years. Most characteristics of these wheat varieties were rich in variation. The results indicated that the wheat materials have high-genetic diversity in these phenotypic characteristics. As a result, the wheat materials can be effectively distinguished and evaluated according to these DUS testing characteristics. However, there was a little difference in awn color, stem color, and glume color among the 195 wheat varieties. It could be explained that these characteristics were irrelevant to the current-breeding objectives.

According to the analysis, results of 195 varieties based on the sources of different wheat-growing areas, it was found that straw pith and seasonal type had large differences between the northern winter wheat zone (Z1), the north Huanghuai winter wheat zone (Z2), and the south Huanghuai winter wheat zone (Z3). The artificial selection trends of the other 19 visually assessed characteristics were similar in the three ecological zones, which could be grouped into three types except for straw pith in cross-section and seasonal type. Among the eight measured characteristics, wheat varieties in the three zones showed the same trends in flag leaf width, plant height, spikelet number per ear, and grain volume weight. There was a declining trend for plant height and an increasing trend for grain volume weight. It has been previously reported that linear regression is suitable for quantitative characteristics to predict changing trends (Jones et al., 2013). Regrettably, the *P-*values between the measured characteristics and the fitted linear equations did not reach a very significant level in this study, except for the trend of grain volume weight for varieties in the south Huanghuai winter wheat zone. We speculate that this may be related to the number of varieties in this study, so other approved wheat varieties remain to be tracked.

Previous studies have shown that abundant waxes on the plant in wheat can improve the resistance to drought (Elham et al., 2012; Dai et al., 2016; Liu et al., 2017) and other biotic and abiotic stresses (Koch et al., 2006; Nawrath, 2006; Yeats and Rose, 2013). The waxes can also improve photosynthesis, reduce transpiration, improve water use efficiency, and ultimately improve grain yield and biological yield (Wang et al., 2015; Xu et al., 2016; Yang et al., 2018; Liu et al., 2020a). Therefore, the wax intensity on flag leaf, stem, ear, and other organs can be used as an indicator for drought-resistant and stress-resistant wheat cultivars. The results showed that the wax on flag leaf sheath and stem showed a gradually increasing trend over time (Figure 3), which suited the needs for drought and stress resistance of wheat. Among the components of wheat yield, fertile ears per hectare have the highest correlation with the yield, followed by 1,000-grain weight (Shi et al., 2009). Studies have shown that traits such as ear length, number of spikelets per ear, number of grains per ear, and volume weight are positively correlated with wheat yield (Zhou et al., 2007; Sadras and Rebetzke, 2013). Reducing the number of sterile spikelets per ear can increase the number of grains per spike, and thus, increase wheat yield. Similarly, it has been reported that plant height has a significant correlation with wheat yield, and reducing plant height can improve harvest index and enhance lodging resistance (Rebetzke and Richards, 2000; Rebetzke et al., 2011). But an unrestricted reduction in plant height can also limit the improvement in biological yield, so both plant height overabundance and overweigh can cause a reduction in yield. The characteristics we used in this study were all derived from the DUS test guidelines and the yield was not measured directly. According to the changing trend of tested characteristics, the length and width of flag leaf, the number of grains per ear, and the grain volume weight of the certified varieties showed an overall upward trend, while the plant height decreased gradually. Our results were consistent with those from studies of cultivars conducted in the previous studies (Donmez et al., 2001; Brancourt-Hulmel et al., 2003; Matus et al., 2012; Song et al., 2013; Liu et al., 2020b). Therefore, it was speculated that wheat yield would be increased by adjusting the plant height, reducing the number of sterile spikelets per ear, and increasing the number of grains per ear, which was in accordance with the mainstream goal of current breeding.

## Conclusion

In this study, 195 wheat varieties in the Huang-Huai-Hai region were used to study their artificial selection trend evaluated by thirty-two DUS testing characteristics. From the perspective of testing for new wheat variety, awn color, stem color, and glume color, is contributive in enriching the genetic diversity of varieties. The straw pith in cross-section and seasonal type had great differences between the three wheat-growing areas under the artificial selection. For the measured characteristics, flag leaf width, plant height, and sterile spikelet number per ear were more closely related to ecological fitness than other measured characteristics. The cluster analysis indicated that the DUS testing characteristics can effectively cluster materials from the same or similar areas, materials from the same approved period, and materials with closer kinship in the same clade. Our results in this study could provide guidance for screening similar varieties and determining the distinctness of applied varieties in DUS testing.

## Data Availability Statement

The original contributions presented in the study are included in the article/Supplementary Material, further inquiries can be directed to the corresponding author/s.

## Author Contributions

HZ and RL designed the research and corrected the manuscript. LW performed the main analyses, prepared figures, and wrote the manuscript. YZ helped in data analysis and revised the manuscript. LD and HL conducted experiments and collected data. MW and HW performed the experiments and supervised the study. All the authors read and approved the final version of the manuscript.

## Funding

This work was supported by grants from the Study on Molecular Marker Assisted Evaluation of Distinctiveness, Uniformity, and Stability of New Wheat Varieties (2015YQN14), the Funds for DUS Testing and Maintenance of Known Variety Library in 2021, and the efficient and accurate detection of crop varieties and seeds (CXGC2022C01).

## Conflict of Interest

The authors declare that the research was conducted in the absence of any commercial or financial relationships that could be construed as a potential conflict of interest.

## Publisher's Note

All claims expressed in this article are solely those of the authors and do not necessarily represent those of their affiliated organizations, or those of the publisher, the editors and the reviewers. Any product that may be evaluated in this article, or claim that may be made by its manufacturer, is not guaranteed or endorsed by the publisher.
